# 30 Jahre Deutsche Einheit: Errungenschaften und verbliebene Unterschiede in der Mortalitätsentwicklung nach Alter und Todesursachen

**DOI:** 10.1007/s00103-021-03299-9

**Published:** 2021-03-25

**Authors:** Pavel Grigoriev, Markéta Pechholdová, Michael Mühlichen, Rembrandt D. Scholz, Sebastian Klüsener

**Affiliations:** 1 Bundesinstitut für Bevölkerungsforschung, Friedrich-Ebert-Allee 4, 65185 Wiesbaden, Deutschland; 2 Max-Planck-Institut für demografische Forschung, Rostock, Deutschland; 3 Wirtschaftsuniversität, Prag, Tschechien; 4 Berliner Institut für Sozialforschung, Berlin, Deutschland; 5 Demografisches Forschungszentrum, Vytautas-Magnus-Universität, Kaunas, Litauen

**Keywords:** Lebenserwartung, Deutsche Wiedervereinigung, Ostdeutschland, Westdeutschland, Dekompositionsanalyse, Life expectancy, German reunification, East Germany, West Germany, Decomposition analysis

## Abstract

**Hintergrund:**

Durch die deutsche Teilung wurden 2 kulturell sehr ähnliche Bevölkerungen sehr unterschiedlichen sozioökonomischen Bedingungen ausgesetzt, die sich nach 1989 wieder anglichen. Der Einfluss von Gesundheitsversorgung und Lebensumständen auf Sterblichkeitsunterschiede kann besser erfasst werden, wenn kulturelle Erklärungen weitgehend ausgeblendet werden können.

**Ziel der Arbeit:**

Die Arbeit wertet harmonisierte Todesursachendaten erstmals detailliert nach Alter aus. Hierdurch kann aufgezeigt werden, welche Alter bzw. Geburtsjahrgänge besonders stark durch die deutsche Teilung und Wiedervereinigung in ihrer Mortalität beeinflusst wurden und auf welche Todesursachen dies zurückzuführen ist.

**Material und Methoden:**

Die deutschen Todesursachenstatistiken wurden einem international standardisierten Harmonisierungsverfahren unterzogen, um Unterschieden und Brüchen in der Todesursachencodierung Rechnung zu tragen. Die Daten wurden mit Dekompositionsmethoden analysiert.

**Ergebnisse:**

In den 1980er-Jahren stiegen die Ost-West-Unterschiede stark an, da Westdeutschland gerade in höheren Altern deutlich höhere Rückgänge bei der kardiovaskulären Mortalität erzielen konnte. Nach 1989 konnte Ostdeutschland in vielen Bereichen zum Westen aufholen. Dies gilt besonders für ältere Personen und Frauen, während gerade bei den stark von der ostdeutschen Transformationskrise betroffenen männlichen Geburtsjahrgängen (1950–1970) noch heute Ost-West-Unterschiede sichtbar sind.

**Diskussion:**

Die geringere Lebenserwartung der ostdeutschen Bevölkerung Ende der 1980er-Jahre war primär durch Rückstände bei der kardiovaskulären Revolution bedingt. Die noch heute bestehenden Unterschiede sind eher Spätfolgen der ostdeutschen Transformationskrise als direkte Spätfolgen der Teilung.

## Abstract (en)

**Background:**

During the German division, two culturally very similar populations were exposed to very disparate socioeconomic conditions, which converged again after 1989. The impact of healthcare and life circumstances on mortality differences can better be estimated when cultural explanations are widely neglectable.

**Objectives:**

For the first time, we analyse harmonised cause-of-death data explicitly by age. Hereby, we can show which ages or birth cohorts were particularly affected by German division and reunification in their mortality and to which causes of death this is attributable.

**Materials and methods:**

We harmonised the German cause-of-death statistics by applying an internationally standardised harmonisation process to account for differences and breaks in cause-of-death coding practices. We analysed the data using decomposition methods.

**Results:**

During the 1980s, east-west disparities were increasing as progress in the reduction of cardiovascular mortality was much stronger in West Germany, notably at older ages. After 1989, East Germany was able to catch up to the west in many areas. This is especially true for elderly persons and women, while east-west disparities are still visible today, particularly among male adult cohorts (1950–1970) strongly affected by the East German transition crisis.

**Conclusions:**

The lower life expectancy of the East German population in the late 1980s was primarily caused by a slower pace of the cardiovascular revolution. The remaining present-day disparities are rather an aftermath of the East German transition crisis than direct aftereffects of the division.

## Einleitung

Die deutsche Wiedervereinigung im Jahr 1990 liegt nun 3 Jahrzehnte zurück. Aber Folgen der Teilung und der ostdeutschen Transformationskrise nach der Wiedervereinigung wirken sich zum Teil bis heute auf Ost-West-Disparitäten in der Sterblichkeit und im Gesundheitsverhalten aus [1–5]. Nichtsdestotrotz sind die Ost-West-Unterschiede bei der durchschnittlichen Lebenserwartung deutlich gesunken. Ostdeutsche Frauen verzeichneten 1990 eine Lebenserwartung von 76,3 Jahren bei Geburt – 2,7 Jahre weniger als westdeutsche Frauen (79,0; alle Lebenserwartungsdaten: Human Mortality Database^1^). Bei den Männern war die Differenz zwischen Ost- (69,2 Jahre) und Westdeutschland (72,6 Jahre) mit 3,4 Jahren noch größer. Als Folge des in den 1990er-Jahren einsetzenden Angleichungsprozesses bestehen auf Basis der aktuellsten Daten für 2017 bei Frauen nur minimale Ost-West-Unterschiede in der Lebenserwartung (Ost: 83,32 Jahre; West: 83,28 Jahre). Bei Männern ist dagegen immer noch eine Lücke von mehr als einem Jahr festzustellen (Ost: 77,66 Jahre; West: 78,81 Jahre), wobei sich diese Differenz seit 2002 kaum verändert hat.

Die allgemeinen Tendenzen in den Sterblichkeitsunterschieden zwischen Ost- und Westdeutschland während und nach der deutschen Teilung wurden bereits in zahlreichen Veröffentlichungen behandelt [6–9]. Eine differenzierte Betrachtung der Auswirkungen der deutschen Teilung anhand der Todesursachenstatistik sah sich dagegen lange mit der Herausforderung konfrontiert, dass keine harmonisierten und vergleichbaren Datenreihen zur Verfügung standen [7, 10–14]. So existierten in der DDR und in Westdeutschland unterschiedliche Praktiken bei der Codierung von Todesursachen, die auch über die Zeit nicht einheitlich waren [15]. Die Brüche in den Zeitreihen hängen mit Übergängen zwischen einzelnen Todesursachenklassifikationen sowie mit der Übernahme westdeutscher Codierungsstandards in Ostdeutschland im Zuge der Wiedervereinigung zusammen [3]. Im Rahmen eines Kooperationsprojektes des Max-Planck-Instituts für demografische Forschung (MPIDR) und des französischen Institut national d’études démographiques (INED) wurden diese Datenreihen harmonisiert. Die harmonisierten Daten erlauben uns nun, einen detaillierten Blick auf die Auswirkungen und Folgen der deutschen Teilung auf Sterblichkeitsunterschiede zwischen Ost- und Westdeutschland zu werfen.

In vorangegangenen Publikationen wurden anhand dieser Daten allgemeine Tendenzen bei der Entwicklung der Todesursachen in Ost- und Westdeutschland für die Gesamtbevölkerung beleuchtet [2, 3]. Hier präsentieren wir eine weiterführende Analyse mit detaillierten Altersklassen und einem erweiterten Untersuchungszeitraum von 1980 bis 2017. Die erste Forschungsfrage ist, wie sich Ost-West-Unterschiede in der Sterblichkeit differenziert nach Geschlecht und Alter vor und nach der Wiedervereinigung entwickelt haben und welche Todesursachen maßgeblich dafür verantwortlich waren. Dies erlaubt Rückschlüsse auf Faktoren, welche einen Einfluss auf Ost-West-Unterschiede in der Sterblichkeit hatten bzw. weiterhin haben. Die zweite Forschungsfrage ist, inwieweit sich die Auswirkungen der Wiedervereinigung auf die todesursachenspezifische Sterblichkeit in Ostdeutschland nach Alter bzw. Generation unterscheiden.

Im Folgenden geben wir zunächst einen Überblick über den thematischen Hintergrund, erklären anschließend die verwendeten Daten und Methoden, um dann die Ergebnisse der Arbeit zu beschreiben und zu diskutieren. Abschließend ziehen wir ein Fazit.

## Hintergrund

Vergleichende Forschung zu den Determinanten von Sterblichkeitsunterschieden zwischen Bevölkerungen unterliegt der Herausforderung, dass oft vielerlei Faktoren für bestehende Differenzen verantwortlich sind. Hierzu zählen Unterschiede bei der Zusammensetzung der betrachteten Bevölkerungen (Altersstruktur, Bildung, kulturelle Prägung) wie auch kontextuelle Faktoren (Gesundheitssystem, ökonomischer Entwicklungsstand und Krisen, ökologische Bedingungen etc.). Diese stehen auch in Wechselwirkung zueinander und waren über die Zeit von unterschiedlicher Dominanz [16, 17]. Ist etwa Fettleibigkeit oder der Konsum von Zigaretten in einer Bevölkerung weitverbreitet, hat dies als kontextueller Einfluss häufig Auswirkungen auf das Gesundheitsverhalten von Individuen in nachfolgenden Generationen. Auch kann die Bevölkerungszusammensetzung einen Einfluss darauf haben, welche potenziell gesundheitsbeeinflussenden Verhaltensweisen gesellschaftlich toleriert bzw. geächtet werden.

Um den Einfluss bestimmter Faktoren besser einschätzen zu können, wird in der komparativen Mortalitätsforschung häufig auf *natürliche Experimente* zurückgegriffen, in welchen ähnliche Bevölkerungen unterschiedlichen Bedingungen ausgesetzt werden. Die deutsche Teilung zwischen 1949 und 1990 wird von vielen Forschenden als ein solches natürliches Experiment angesehen [18–22]. Zwei kulturell sehr ähnliche Bevölkerungen wurden in dieser Periode in der westdeutschen Bundesrepublik und der ostdeutschen DDR sehr unterschiedlichen politischen und sozioökonomischen Rahmenbedingungen ausgesetzt. Diese hatten auch Differenzen im Gesundheitssystem zur Folge, welche ab den 1970er-Jahren zu starken Disparitäten bei der Lebenserwartung beitrugen [23–25]. Damals konnte die DDR wie auch andere osteuropäische Staaten im Zuge der sogenannten *kardiovaskulären Revolution* nicht mit dem Westen Schritt halten, da sie angesichts eingeschränkter Ressourcen in einem weitaus geringeren Ausmaß die hierfür nötigen Investitionen in die Gesundheitsinfrastruktur vornehmen konnte [6, 7, 12, 17, 19, 26, 27]. Die kardiovaskuläre Revolution beschreibt die dauerhafte Reduktion der kardiovaskulären Mortalität sowohl durch grundlegende Veränderungen verhaltensbedingter Risikofaktoren als auch durch Fortschritte der Medizintechnologie und Krankheitsprävention [26]. Mit ihr konnten gerade im höheren Alter ab 60 Jahren Fortschritte beim Sterblichkeitsrückgang verzeichnet werden [17]. Das Gesundheitsversorgungssystem der DDR fokussierte sich aufgrund der eingeschränkten Ressourcen dagegen eher auf die Bevölkerung im erwerbsfähigen Alter [12].

Von der deutschen Wiedervereinigung konnten im Osten ältere Personen besonders profitieren. Hierzu trug die Übernahme des westdeutschen Rentensystems in Ostdeutschland bei [11, 20, 28]. Da es in der DDR offiziell keine Arbeitslosigkeit gab und die Frauenerwerbsbeteiligung hoch war, wiesen Anfang der 1990er-Jahre viele ostdeutsche Rentnerinnen und Rentner lange, ununterbrochene Erwerbsbiografien auf. Diese sicherten ihnen relativ hohe Renten, für Frauen sogar höhere Renten als im Westen [29, 30]. Außerdem wurden große Anstrengungen unternommen, die technischen Standards in ostdeutschen Krankenhäusern schnell an das Westniveau anzupassen; Gleiches galt für die Ausstattung von Notfallrettungsdiensten [12]. Diese Fortschritte wirkten sich insbesondere auf die Behandlung von (akuten) Krankheiten des Kreislaufsystems sehr positiv aus. Da derartige Krankheiten im höheren Alter deutlich häufiger auftreten, konnten gerade in diesem Alter Sterblichkeitsrückgänge erzielt werden [7, 9, 12, 18, 21, 22]. Zu der schnellen Reduktion der Ost-West-Unterschiede bei den älteren Frauen trug ebenfalls bei, dass die in Westdeutschland in den 1940er- bis 1960er-Jahren geborenen Frauenkohorten einen höheren Anteil an Raucherinnen aufwiesen, was in diesen Kohorten insbesondere nach 1990 zu einem West-Ost-Gefälle in der Lungenkrebsmortalität führte [5].

Ein anderes Bild ergibt sich für die ostdeutschen Generationen, die sich zum Zeitpunkt der Wiedervereinigung und der anschließenden Transformationskrise im erwerbsfähigen Alter befanden. Sie waren in dieser Phase ökonomischer Umwälzungen und Unsicherheiten in einem besonderen Maße von struktureller Arbeitslosigkeit betroffen [31–35]. Der daraus resultierende psychosoziale Stress spiegelte sich auch in einem Anstieg der alkoholbedingten Sterblichkeit gerade bei Männern ab 30 Jahren wider [12, 19, 31]. Obgleich sich die Lage auf dem ostdeutschen Arbeitsmarkt in den letzten Jahren deutlich aufgehellt hat, so hat die Transformationskrise Spuren in vielen Erwerbsbiografien hinterlassen. Gerade die zwischen 1946 und 1970 geborenen Ostdeutschen erlebten vergleichsweise häufig Phasen mit geringen Löhnen und längere Unterbrechungen durch Arbeitslosigkeit. Dies spiegelt sich, besonders bei Männern, zunehmend auch in relativ geringen Renten im Vergleich zum Westen wider [29, 30]. Diese Lücke bleibt wahrscheinlich noch einige Jahrzehnte bestehen. Hinzu kommt, dass Rauchen im Osten bei den ab den späten 1970ern geborenen Kohorten stärker verbreitet ist als im Westen [1, 5]. Jenes gilt insbesondere für Frauen. Die unsteten Erwerbsbiografien und das Rauchverhalten in den jungen und mittleren Kohorten könnten dazu führen, dass in der Zukunft neue Ost-West-Disparitäten in der Sterblichkeit auch im höheren Alter auftreten [1, 5].

## Daten und Methoden

Unsere Analysen beruhen auf den Todesursachenstatistiken, die von den Statistischen Ämtern des Bundes und der Länder sowie der Zentralstelle für Statistik (Statistisches Amt der DDR) erstellt wurden [3]. Da Ost- und Westberlin mit der Einführung von ICD-10 seit 1998 nicht mehr getrennt in der Todesursachenstatistik erhoben werden, rechnen wir ab 1998 auch Westberlin dem Osten zu. In Bezug auf Zeitreihenbrüche durch Änderungen bei Codierungspraktiken stellte der Übergang von der 9. zur 10. Revision der *Internationalen Klassifikation der Krankheiten* (ICD) eine Herausforderung dar. Diesbezüglich wurden die Daten einem international standardisierten Harmonisierungsverfahren unterzogen. Die Standardisierung erfolgte mit einer verkürzten Liste von 186 Todesursachen („186-Liste“), welche auf Basis von Erfahrungen bei der Standardisierung von Todesursachenstatistiken in verschiedenen Ländern erstellt wurde [36]. Sie hat 5 Ziele: (1) Minimierung von Brüchen beim Übergang von ICD‑9 auf ICD-10; (2) Maximierung der internationalen Vergleichbarkeit; (3) Kompatibilität mit anderen häufig verwendeten Klassifizierungen; (4) Sensibilität für den jeweiligen epidemiologischen Kontext; (5) Übernahme der Dokumentation soll weitgehend möglich sein.

Ein Problem war, dass in der DDR im Zeitraum 1976–1989 einige Todesursachen als politisch sensibel galten und daher für die offiziellen Statistiken umcodiert wurden. Hierzu zählten bestimmte Krankheiten der Verdauungsorgane und Suizide [7, 35, 37]. Durch Archivrecherchen war es uns möglich, den Zugang zu diesen während der DDR-Zeit unter Verschluss gehaltenen Daten zu bekommen, um die notwendigen Ergänzungen und Korrekturen an den veröffentlichten Statistiken vornehmen zu können.

Eine große Herausforderung war auch, Differenzen aufgrund von Unterschieden in den Codierungspraktiken zwischen Ost- und Westdeutschland zu beseitigen. So waren bei der Einführung der westdeutschen Codierungspraktiken in Ostdeutschland Anfang der 1990er-Jahre bei einigen Todesursachen starke Sprünge zu beobachten, die nicht durch Effekte der Wendezeit begründet werden konnten. So stieg etwa der Anteil der Todesfälle durch Neubildungen aufgrund der veränderten Einordnung beim Grundleiden schlagartig um 10 % (siehe auch [3, 11]); ebenso waren bei Krankheiten des Kreislaufsystems erhebliche Trendbrüche zu beobachten. Diese mussten identifiziert und berichtigt werden. Die für die Analyse verwendeten Todesursachenkategorien der 186-Liste und die entsprechenden ICD-10-Codes sind in Tab. 1 aufgeführt. Insgesamt betrachten wir 6 Obergruppen von Todesursachen: Infektions- und Atemwegskrankheiten, Neubildungen, Krankheiten des Kreislaufsystems, Krankheiten des Verdauungssystems, äußere Ursachen und andere Ursachen. Zudem untersuchen wir die Entwicklung von Todesfällen an ausgewählten Krankheiten des Kreislaufsystems (akuter Myokardinfarkt, chronische ischämische Herzkrankheit, zerebrovaskuläre Krankheiten) und anderen Todesursachen mit Relevanz für den Umbruchprozess im Kontext der Wiedervereinigung (chronische Leberkrankheiten und Zirrhosen, Transportmittelunfälle, Suizide, Alkoholmissbrauch). Es ist geplant, die korrigierten Datenserien in der neuen *Human Cause-of-Death Database* [38] zu veröffentlichen.

**Table 1:** 

	ICD-10	186-Liste
*Infektions- und Atemwegskrankheiten*	A00–B99, J00–J99	1–25,101–115
*Neubildungen*	C00–D48	26–55
*Krankheiten des Kreislaufsystems*	I00–I99, G65	77–100
Akuter Myokardinfarkt	I21–I22	84
Chronische ischämische Herzkrankheit	I20, I25	86
Zerebrovaskuläre Krankheiten	I60–I69, G45	93–95
*Krankheiten des Verdauungssystems*	K00–K99	116–124
Chronische Leberkrankheiten und Zirrhosen	K70, K73, K74	122
*Äußere Ursachen*	V00–Y99	156–186
Transportmittelunfälle	V02–V04, V09, V12–V14, V20–V79	156
Suizide	X60–X84	168–172
*Andere Ursachen*	Alle anderen Ursachen	Alle anderen Ursachen
Alkoholmissbrauch	F10	65

Um detailliert zu eruieren, inwieweit die deutsche Teilung und die ostdeutsche Transformationskrise nach der Wiedervereinigung Ost-West-Differenzen in der Mortalität beeinflusst haben, führen wir Dekompositionsanalysen durch [39]. Diese in demografischen Studien häufig angewandte Methode [40] ermöglicht, ein tieferes Verständnis darüber zu gewinnen, welche Todesursachen und Altersgruppen zu verschiedenen Zeitpunkten besonders stark zu Ost-West-Unterschieden in der Lebenserwartung beigetragen haben. Hierbei standardisieren wir die Daten für Ostdeutschland gegenüber den Daten für Westdeutschland, um aufzuzeigen, wie der Osten gegenüber dem Westen abweicht. Außerdem betrachten wir ausgewählte Todesursachentrends für Ostdeutschland anhand standardisierter Sterberaten (SDR), welche gegenüber rohen Raten den Vorteil bieten, dass Effekte durch Unterschiede und Veränderungen in der Altersstruktur herausgerechnet werden [40]. Hierbei haben wir auf die Europäische Standardbevölkerung der Weltgesundheitsorganisation (ESP 1976) zurückgegriffen. Von Interesse sind für uns insbesondere (1) die Trends in der todesursachenspezifischen Sterblichkeit im letzten Jahrzehnt der Teilung, (2) die Präsenz bzw. Abwesenheit von Trendveränderungen zu Beginn der Umbruchphase um 1990 und (3) die weitere Entwicklung in den Jahrzehnten nach der Wiedervereinigung.

## Ergebnisse und Diskussion

Unsere Analyse der Todesursachenstatistiken beginnen wir zunächst mit der Beschreibung einiger wichtiger Trends. Bei den dominierenden Todesursachen durch Krankheiten des Kreislaufsystems bestanden bei beiden Geschlechtern in den 1980er-Jahren erhebliche Ost-West-Unterschiede, die besonders bei Frauen stark ausgeprägt waren. Die auf Basis unserer harmonisierten Daten ermittelten standardisierten Sterberaten (SDR) lagen bei Männern um 1990 bei 462 in West- und 698 in Ostdeutschland, und bei Frauen bei 308 (West) bzw. 463 (Ost). Diese Differenzen werden in der wissenschaftlichen Diskussion zum großen Teil auf die beschriebene Situation zurückgeführt, dass die DDR in der kardiovaskulären Revolution bei der technischen Ausstattung des Gesundheitswesens mit Westdeutschland nicht mithalten konnte [7, 12, 17]. Allerdings waren bereits in den 1980er-Jahren leichte Angleichungstendenzen erkennbar, die sich insbesondere nach 1995 weiter verstärkten (siehe auch [2, 3]). Selbst im jüngsten Jahr des Untersuchungszeitraums (2017) sind die SDRs durch Krankheiten des Kreislaufsystems im Osten noch etwas höher als im Westen, sowohl bei Männern mit 248 (Ost) gegenüber 216 (West) als auch bei Frauen mit 162 (Ost) gegenüber 145 (West). Die verbliebenen Unterschiede werden in der Literatur einerseits mit der Distanz zur medizinischen Versorgung in besonders peripheren Regionen im Osten erklärt, die gerade bei Herzinfarkten und Schlaganfällen eine Rolle spielt; andererseits – gerade bei Männern – mit der größeren Verbreitung von Risikofaktoren wie Rauchen und Alkoholmissbrauch [12, 14, 21, 24].

Bei Neubildungen verzeichnete die DDR dagegen bei beiden Geschlechtern geringere SDRs, was zum Teil auch der Tatsache geschuldet war, dass dort kardiovaskuläre Todesursachen dominanter waren. Nach 1990 sehen wir bei Männern und Frauen sehr unterschiedliche Entwicklungen. Bei Männern verzeichnet der Osten seit 2000 höhere SDRs als der Westen. 2017 lagen diese im Osten bei 213 und im Westen bei 192; bei Frauen hingegen haben sich die Raten auf einem Niveau von etwa 130 angeglichen. Wie bereits weiter oben diskutiert wurde, zeigen aber Forschungsergebnisse auf, dass bei jüngeren ostdeutschen Frauenkohorten in den letzten Jahrzehnten starke Anstiege beim Anteil der Raucherinnen verzeichnet wurden, die heute über den Anteilen bei Frauen im Westen liegen [5]. Daraus wird abgeleitet, dass in Zukunft neue Ost-West-Disparitäten bei Todesfällen durch Neubildungen wie Lungenkrebs bei Frauen auftreten können [1]. Bei Infektions- und Atemwegskrankheiten sind die SDRs in beiden Landesteilen relativ niedrig, sodass heute nur geringe absolute Ost-West-Unterschiede bestehen, sowohl bei Männern mit 60 (Ost) bzw. 65 (West) als auch bei Frauen mit 32 (Ost) bzw. 40 (West).

Bei Krankheiten des Verdauungssystems und äußeren Todesursachen wurden im Osten fast während der gesamten Beobachtungsperiode höhere Todesraten verzeichnet. Dabei stiegen die Ost-West-Unterschiede in der Umbruchphase um 1990 sprunghaft an, was in der Literatur u. a. auf einen erhöhten stressbedingten Konsum von Alkohol und den plötzlich erheblich verbesserten Zugang zu stark motorisierten Fahrzeugen zurückgeführt wird [41–43]. Auf diese Aspekte gehen wir weiter unten im Detail ein. Bei Männern verzeichnet der Osten bis heute höhere Sterberaten bei Krankheiten des Verdauungssystems (SDR Ost: 46; SDR West: 33).

**Figure 1:**
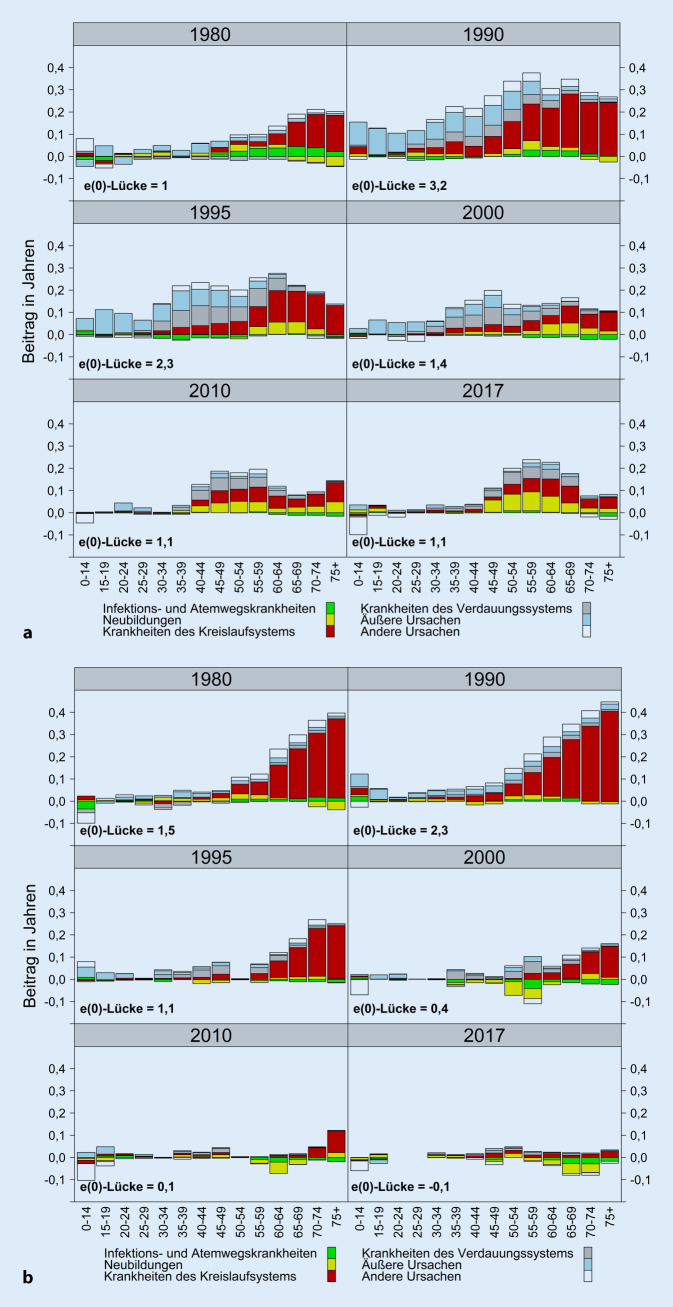

Einen genaueren Einblick in den Beitrag spezifischer Todesursachen in bestimmten Altersgruppen zur Ost-West-Lücke in der Lebenserwartung bei Geburt bieten unsere Dekompositionsanalysen. Deren Ergebnisse werden in den Abb. 1a, b dargestellt, welche die Differenzen der Lebenserwartung zwischen Ost und West standardisiert nach Altersklassen und Todesursachen aufzeigen. Wir konzentrieren uns in dieser Darstellung auf die 6 Obergruppen der Todesursachen. Westdeutschland dient als Referenz, d. h., Werte oberhalb von 0 bedeuten für den Osten eine höhere Sterblichkeit, Werte unterhalb von 0 eine niedrigere Sterblichkeit in der jeweiligen Alters- und Todesursachengruppe. Wie beide Abbildungen veranschaulichen, waren es 1980 insbesondere die Krankheiten des Kreislaufsystems im hohen Alter, die zu den Ost-West-Unterschieden bei der Lebenserwartung beitrugen. Dies galt gerade für Frauen. Im Wiedervereinigungsjahr 1990 ergab sich für Frauen noch ein ähnliches Bild wie 1980. Allerdings war in jüngeren Altersgruppen eine etwas größere Lücke zwischen Ost- und Westdeutschland festzustellen, die überwiegend durch eine erhöhte Sterblichkeit aufgrund von äußeren Ursachen wie etwa Verkehrsunfälle zustande kam. Bei Männern hatte sich in den 1980ern die Lücke zum Westen in allen Altersgruppen vergrößert. In den hohen Altersklassen waren dabei weiter Krankheiten des Kreislaufsystems maßgeblich für die Mortalitätsunterschiede verantwortlich, während in den Altersklassen unter 50 Jahren gerade Sterbefälle durch äußere Ursachen wie Verkehrsunfälle und Krankheiten des Verdauungssystems zu den Ost-West-Differenzen beitrugen. Die Ost-West-Unterschiede sind bei Männern vor allem in den 1990er-Jahren zurückgegangen, besonders deutlich bei Krankheiten des Kreislaufsystems. Danach gab es nur noch leichte Reduktionen, speziell im Kindes- und jungen Erwachsenenalter durch einen Rückgang von äußeren Todesursachen bei gleichzeitiger Verschiebung der Sterblichkeit an Verdauungskrankheiten in höhere Altersgruppen.

In der jüngeren Zeit konzentrieren sich die Unterschiede zwischen ost- und westdeutschen Männern vor allem auf die Geburtskohorten 1950 bis 1970. Diese waren zum Zeitpunkt der Wiedervereinigung und in der nachfolgenden Transformationskrise im jungen Erwachsenenalter, einem Alter, in dem in der Regel die Etablierung auf dem Arbeitsmarkt erfolgt. Die aktuelle Übersterblichkeit ostdeutscher Männer dieser Geburtsjahrgänge beruht weitgehend auf erhöhten Sterberaten bei Kreislauf- und Verdauungskrankheiten sowie in zunehmender Weise bei Neubildungen. Bei Krankheiten des Verdauungssystems handelt es sich hier überwiegend um Folgen eines erhöhten Alkoholkonsums, der in der Literatur mit erhöhtem psychosozialen Stress aufgrund der ökonomischen Unsicherheiten im Zuge der ostdeutschen Transformationskrise in Verbindung gebracht wird [12, 32, 35, 43]. Forschungsergebnisse zeigen, dass gerade männliche Arbeitslose einen schlechteren allgemeinen Gesundheitszustand aufweisen [33, 34, 44, 45] und in beiden Landesteilen ein deutlich höheres Sterberisiko als nichtarbeitslose Personen haben [31, 32]. Die Ursache für die erhöhte Sterblichkeit bei Männern im Osten könnte daher u. a. durch den höheren Anteil von Männern mit unterbrochenen Erwerbsbiografien bedingt sein. In den Geburtsjahrgängen nach 1970 sind die Sterberaten aufgrund des jungen Alters sowohl im Osten als auch im Westen sehr gering und die Unterschiede dementsprechend klein.

Bei Frauen glichen sich die Disparitäten nach der Einheit schneller an. Bereits 1995 waren die Ost-West-Unterschiede um etwa die Hälfte zurückgegangen. In den Jahren danach sank die Übersterblichkeit weiter und konzentrierte sich zunehmend auf das hohe Alter und Krankheiten des Kreislaufsystems. Inzwischen lassen sich keine gravierenden Unterschiede mehr zwischen ost- und westdeutschen Frauen feststellen. Bei Neubildungen weist in vielen Altersgruppen der Westen eine höhere Sterblichkeit auf. Dies gilt insbesondere für die Geburtsjahrgänge 1942–1950, in denen auch der Anteil der Raucherinnen höher ist als im Osten [5]. Im Kindesalter (0 bis unter 15 Jahre) ist die Sterblichkeit im Westen für beide Geschlechter höher als im Osten.

**Figure 2:**
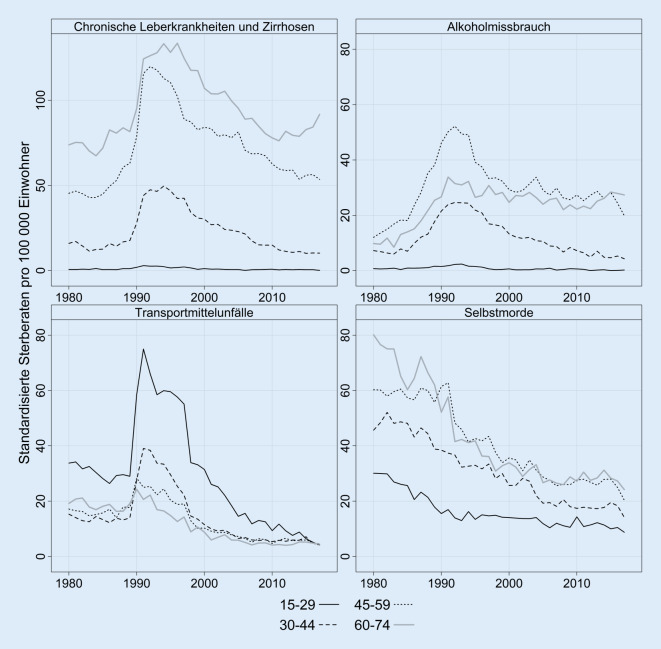

**Figure 3:**
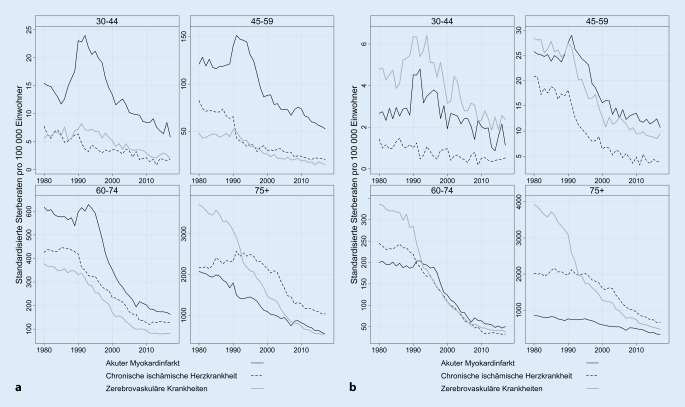

In den Abb. 2 und Abb. 3 zeigen wir für Ostdeutschland die Entwicklung ausgewählter Todesursachen, welche für die Analyse der Auswirkungen der Wende besonders relevant sind [46]. In Abb. 2 fokussieren wir uns auf die ostdeutschen Männer, da sich bei diesen die Transformationskrise besonders stark manifestierte. Bei den Sterbefällen durch chronische Leberkrankheiten, Leberzirrhosen und Alkoholmissbrauch sehen wir ab Ende der 1980er-Jahre einen starken Anstieg. Dieser erreichte in den frühen 1990er-Jahren seinen Höhepunkt und war insbesondere bei Männern ab 30 Jahren festzustellen. Ab Mitte der 1990er-Jahre ging die Mortalität durch diese Ursachen wieder zurück, wobei die Sterblichkeit durch Alkoholmissbrauch bei Männern ab 45 Jahren weiterhin höher ist als in den 1980er-Jahren. Dies sind wahrscheinlich Spätfolgen der Transformationskrise. In der Altersgruppe der 60- bis 74-Jährigen ist in den letzten 5 Jahren des Untersuchungszeitraums ein erneuter Aufwärtstrend bei chronischen Leberkrankheiten und -zirrhosen sowie in abgeschwächter Form auch bei Alkoholmissbrauch zu verzeichnen.

Bei Verkehrsunfällen kam es 1990 zu einem drastischen Anstieg, der in der Literatur mit der sprunghaften Zunahme der Motorisierung in Ostdeutschland und mit der nicht an das Verkehrsaufkommen angepassten Infrastruktur in Zusammenhang gebracht wird [41, 42]. Dieser Anstieg wurde insbesondere durch junge Altersgruppen getragen und speziell durch Personen im Alter zwischen 15 und 29 Jahren. Bei dieser Todesursache setzte ab Mitte der 1990er-Jahre wieder ein deutlicher Rückgang ein, sodass bei allen betrachteten Altersklassen die Sterberaten heute unter denen vor der Wiedervereinigung liegen. Bei Suiziden ist dagegen kein Anstieg um die Wiedervereinigung erkennbar. Die Raten gingen in allen Altersklassen in den 1980er-Jahren und der ersten Hälfte der 1990er-Jahre stetig zurück und verharren seit Ende der 1990er-Jahre auf sehr niedrigem Niveau (siehe auch [37]).

Für Krankheiten des Kreislaufsystems zeigen wir die Trends sowohl für ostdeutsche Männer (Abb. 3a) als auch für ostdeutsche Frauen (Abb. 3b). Unsere harmonisierten Daten verdeutlichen, dass in den 1980er-Jahren auch in der DDR im höheren Alter ab 60 Jahren Sterblichkeitsrückgänge gerade bei zerebrovaskulären Krankheiten verzeichnet werden konnten (siehe auch [25]). Diese Rückgänge waren nach 1990 noch einmal deutlich stärker als in den 1980er-Jahren. Bei jüngeren Altersgruppen kam es dagegen um 1990 zu einem Anstieg der Sterblichkeit durch akute Myokardinfarkte, wobei dieser Anstieg bei Männern besonders stark ausfiel. Seit Mitte der 1990er-Jahre entwickelt sich die Mortalität in allen Altersgruppen bei allen betrachteten Todesursachen tendenziell rückläufig.

Die hier präsentierten Analysen sind nicht frei von Limitationen. Bezüglich der Qualität der Todesursachenstatistik ist hervorzuheben, dass Tode häufig im Kontext von Multimorbidität auftreten. Hierdurch ist das Grundleiden nicht immer eindeutig bestimmbar. Aufgrund dieser Interpretationsspielräume existieren zum Teil auch regionale Unterschiede in Codierungspraktiken. Des Weiteren werden Todesbescheinigungen nicht immer sorgfältig ausgefüllt, was zu Problemen bei der Bestimmung des Grundleidens bei der Todesursachencodierung führen kann. Eine weitere Herausforderung bei unserer Harmonisierung der Daten war, dass in Ostdeutschland der Übergang von der DDR-Codierungspraxis zu westdeutschen Standards weitgehend undokumentiert blieb [3]. Die implementierten Korrekturen sind aber konsistent mit den Ergebnissen bisheriger Studien [47, 48]. Wir haben keine Hinweise darauf, dass Datenprobleme unsere Hauptschlussfolgerungen beeinträchtigen (siehe auch [3]). Es ist auch wichtig zu betonen, dass Todesursachenstatistiken generell keine direkten Rückschlüsse auf die Morbidität der Bevölkerung zulassen.

## Fazit

Die im Rahmen des Forschungsprojekts harmonisierten Zeitreihen zur todesursachenspezifischen Sterblichkeitsentwicklung eröffnen neue Perspektiven auf das natürliche Experiment der deutschen Teilung und Wiedervereinigung. Außerdem ermöglichen sie die Analyse kurz- und langfristiger Auswirkungen der Transformationskrise in Ostdeutschland auf Sterblichkeitsentwicklungen in den verschiedenen Generationen. Wir konnten darlegen, dass vor allem ältere Personen – und Frauen generell – nach der Wiedervereinigung relativ schnell bei der Lebenserwartung zum Westen aufschließen konnten. Die zügige Verbesserung der medizinischen Versorgung wirkte sich insbesondere bei der Sterblichkeit an kardiovaskulären Krankheiten positiv aus, die vornehmlich im höheren Alter auftreten. Die geringere Lebenserwartung in der DDR in den 1980er-Jahren scheint dementsprechend primär durch Rückstände bei der medizinischen Versorgung bedingt gewesen zu sein. Bei den stark von der ostdeutschen Transformationskrise betroffenen männlichen Kohorten (1950–1970) sind hingegen noch heute Ost-West-Unterschiede etwa bei alkoholbedingten Todesfällen sichtbar. Die nach 1970 geborenen Männer, die zur Wendezeit zum größeren Teil noch nicht auf dem Arbeitsmarkt waren, zeigen dagegen bisher kaum Ost-West-Unterschiede. Diese Kohorten sind jedoch auch noch in einem Alter mit generell geringer Sterblichkeit. Insofern scheinen sich die Nachwirkungen der Transformationskrise derzeit überwiegend auf die Kohorten zu konzentrieren, die als Erwachsene im erwerbsfähigen Alter von den Unsicherheiten betroffen waren.

Die beobachteten Entwicklungen werfen die Frage auf, ob Ost-West-Disparitäten in der Sterblichkeit bald komplett der Vergangenheit angehören. Diesbezüglich ist hervorzuheben, dass die sozioökonomischen Bedingungen im Osten im Durchschnitt weiterhin ungünstiger als im Westen sind. In der Literatur wird dies als ein wesentlicher Faktor für die insbesondere bei Männern immer noch verzeichnete erhöhte Sterblichkeit angesehen [4, 31, 32, 49, 50]. Die lange Phase hoher struktureller Arbeitslosigkeit in Ostdeutschland wird tendenziell in den nächsten Jahrzehnten auch zu steigenden Ost-West-Unterschieden bei der Rentenhöhe beitragen [29, 30], welche wiederum positiv mit der Lebenserwartung assoziiert ist [44, 50]. Weiterhin belegen Studien, dass im Gegensatz zu den älteren Frauen bei den jüngeren Frauen der Anteil der Raucherinnen im Osten deutlich höher als im Westen ist [1, 5]. Daher ist es nicht unwahrscheinlich, dass in der Zukunft Ost-West-Unterschiede bei der Sterblichkeit wieder zunehmen werden. Insofern könnten die Folgen der Teilung deutlich länger in Ost-West-Disparitäten bei der Sterblichkeit sichtbar sein, als die Teilung insgesamt dauerte. Die heute bestehenden Unterschiede sind aber eher Folgen der ostdeutschen Transformationskrise nach der Wiedervereinigung als direkte Folgen der Teilung.

## Footnotes

1 Human Mortality Database des Max-Planck-Instituts für demografische Forschung, Rostock, und der Universität von Kalifornien, Berkeley: https://www.mortality.org. Zugegriffen am 24.08.2020.
